# Impact of post-filter ionized calcium target range on circuit survival and citrate-related complications in pediatric continuous kidney replacement therapy

**DOI:** 10.1007/s00467-026-07440-1

**Published:** 2026-07-08

**Authors:** Muhterem Duyu, Ayşe Aşık

**Affiliations:** Department of Pediatric Intensive Care, Istanbul Goztepe Prof. Dr. Suleyman Yalcin City Hospital, Istanbul, Turkey

**Keywords:** Regional citrate anticoagulation, Continuous kidney replacement therapy, Pediatric critical care, Circuit survival, Hypocalcemia

## Abstract

**Background:**

Regional citrate anticoagulation (RCA) is the preferred strategy for continuous kidney replacement therapy in children; however, the optimal post-filter ionized calcium target remains uncertain. Lower targets may increase anticoagulation but raise citrate exposure and metabolic complications. We aimed to compare anticoagulation efficacy and metabolic safety between a low-target (0.25–0.35 mmol/L) and a high-target (0.30–0.40 mmol/L) post-filter ionized calcium protocol in critically ill children.

**Methods:**

This retrospective cohort study included critically ill children receiving continuous veno-venous hemodiafiltration with citrate as the pre-filter anticoagulation solution at a tertiary pediatric intensive care unit over a 4-year period. A total of 87 patients (42 low-target, 45 high-target) and 154 circuits (71 versus 83) were analyzed. The primary outcome was circuit survival (CS). Secondary outcomes included citrate dose, citrate load, and RCA-related complications. Continuous variables were analyzed using the Mann–Whitney *U* test, and CS was assessed with Cox regression. Linear mixed models evaluated citrate changes, and generalized estimating equations analyzed metabolic outcomes.

**Results:**

Median CS was comparable between groups (49 versus 48 h, *p* = 0.76) as was the survival of clotted circuits (41 versus 40 h, *p* = 0.70) and circuit clotting rates were similar (21.1% versus 24.1%, *p* = 0.70). The low-target group had higher median citrate dose (2.9 versus 2.6 mmol/L, *p* < 0.001), citrate load (0.76 versus 0.72 mmol/kg/h, *p* = 0.03), and more frequent hypocalcemia (17.5% versus 12.9%, *p* = 0.01), metabolic alkalosis (31.9% versus 22.8%, *p* < 0.001), and citrate accumulation (24.5% versus 15.4%, *p* < 0.001). Linear mixed models showed a persistently higher citrate dose and citrate load in the low-target group (all *p* < 0.001). Generalized estimating equations demonstrated increased odds of hypocalcemia (odds ratio 1.47, *p* = 0.01) and citrate accumulation (odds ratio 1.93, *p* < 0.001) in the low-target group.

**Conclusions:**

Raising the target of post-filter ionized calcium from 0.25–0.35 to 0.30–0.40 mmol/L reduced citrate exposure and metabolic complications without compromising CS.

**Trial registration:**

Retrospectively registered.

**Graphical abstract:**

**Figure:**
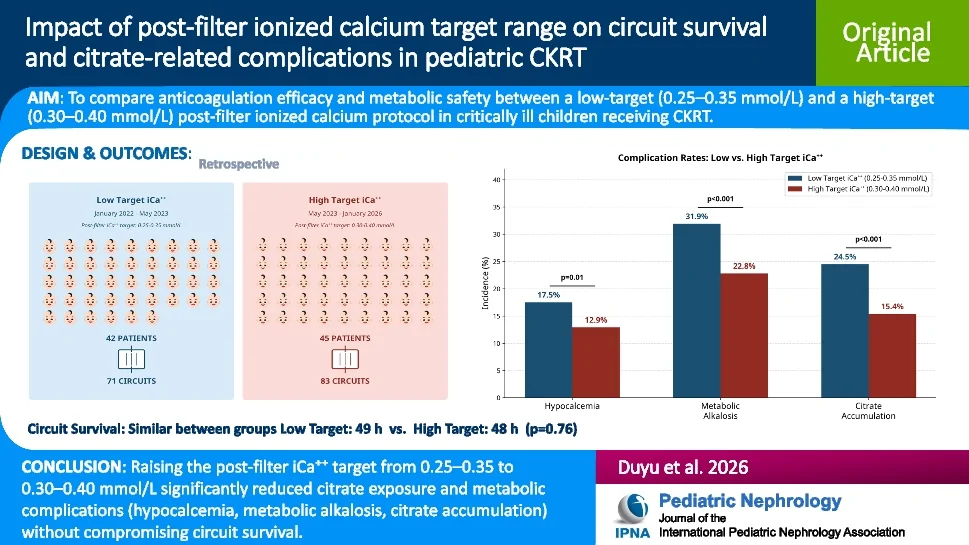
A higher resolution version of the Graphical abstract is available as Supplementary information

**Supplementary Information:**

The online version contains supplementary material available at https://doi.org/10.1007/s00467-026-07440-1.

## Introduction

Continuous kidney replacement therapy (CKRT) is the most commonly used modality of kidney replacement therapy (KRT) in critically ill pediatric patients [1, 2]. The therapeutic effectiveness of CKRT largely depends on hemofilter lifespan, as uninterrupted circuit function is essential for adequate treatment delivery. To prevent circuit clotting and maintain treatment efficacy, effective anticoagulation is required [3]. Compared with systemic unfractionated heparin, regional citrate anticoagulation (RCA) is associated with longer circuit lifespan by reducing treatment downtime [4–11], which is particularly important in pediatric patients with limited circulating blood volume, as it contributes to minimizing blood loss and bleeding-related complications [3]. RCA is increasingly preferred as the anticoagulation strategy for CKRT worldwide for both adult and pediatric CKRT patients [4–11]. Despite its advantages, RCA may be associated with metabolic and electrolyte disturbances, including citrate accumulation (CA), which represents the most clinically significant complication [5, 8]. If left untreated, CA accompanied by severe hypocalcemia may result in cardiovascular collapse [12].

Citrate provides anticoagulation within the extracorporeal circuit by chelating serum ionized calcium (iCa^++^), a key cofactor in multiple steps of the coagulation cascade [13, 14]. The levels of post-filter ionized calcium (pf-iCa^++)^ serve as a significant indicator in the optimization of citrate dosage in RCA [15, 16]. It has been documented that reducing iCa^++^ levels below 0.35 mmol/L optimizes anticoagulation efficacy; therefore, most current protocols typically define target pf-iCa^++^ range as 0.25–0.35 mmol/L [17, 18]. Nonetheless, despite the widespread use of RCA, the relationship between pf-iCa^++^ levels and circuit survival (CS) remains incompletely understood [4, 19–24]. Increasing the targeted pf-iCa^++^ levels may reduce citrate requirements and thereby decrease RCA-related adverse effects; however, it also raises concerns regarding premature circuit clotting. Although a limited number of recent adult studies have reported that higher pf-iCa^++^ targets do not compromise anticoagulation efficacy, no corresponding evaluation has been reported in pediatric population [21, 25].

The primary objective of our study is to evaluate the anticoagulation efficacy (CS until clotting) of two different pf-iCa^++^ targets: a low target (LT) range of 0.25–0.35 mmol/L and a high target (HT) range of 0.30–0.40 mmol/L, in critically ill children undergoing CKRT. Our secondary objective is to evaluate whether a HT-pf-iCa^++^ reduces citrate load and thus decreases side effects associated with RCA.

## Materials and methods

This retrospective cohort study was conducted in a multidisciplinary PICU within a tertiary referral center. The institutional review board, under registration number 2025/0162, granted approval for the research plan. As this was retrospective research, the board decided that informed consent was not necessary.

### Patients and study design

Starting in January 2022, our pediatric intensive care unit (PICU) implemented a protocol for critically ill children undergoing CKRT that involves a low initial citrate infusion dose (CID). For patients weighing 10 kg or less, the initial CID was established at 2.2 mmol/L, while for those over 10 kg, it was set at 2.5 mmol/L. This protocol was applied to patients aged between 28 days and 18 years from January 2022 to January 2026. In May 2023, the protocol was adjusted to raise the pf-iCa^++^ target range from 0.25–0.35 mmol/L to 0.30–0.40 mmol/L. Patients were divided into two groups: LT-pf-iCa^++^ (0.25–0.35 mmol/L) from January 2022 to May 2023 and HT-pf-iCa^++^ (0.30–0.40 mmol/L) from May 2023 to January 2026 (Fig. 1).

**Fig. 1:**
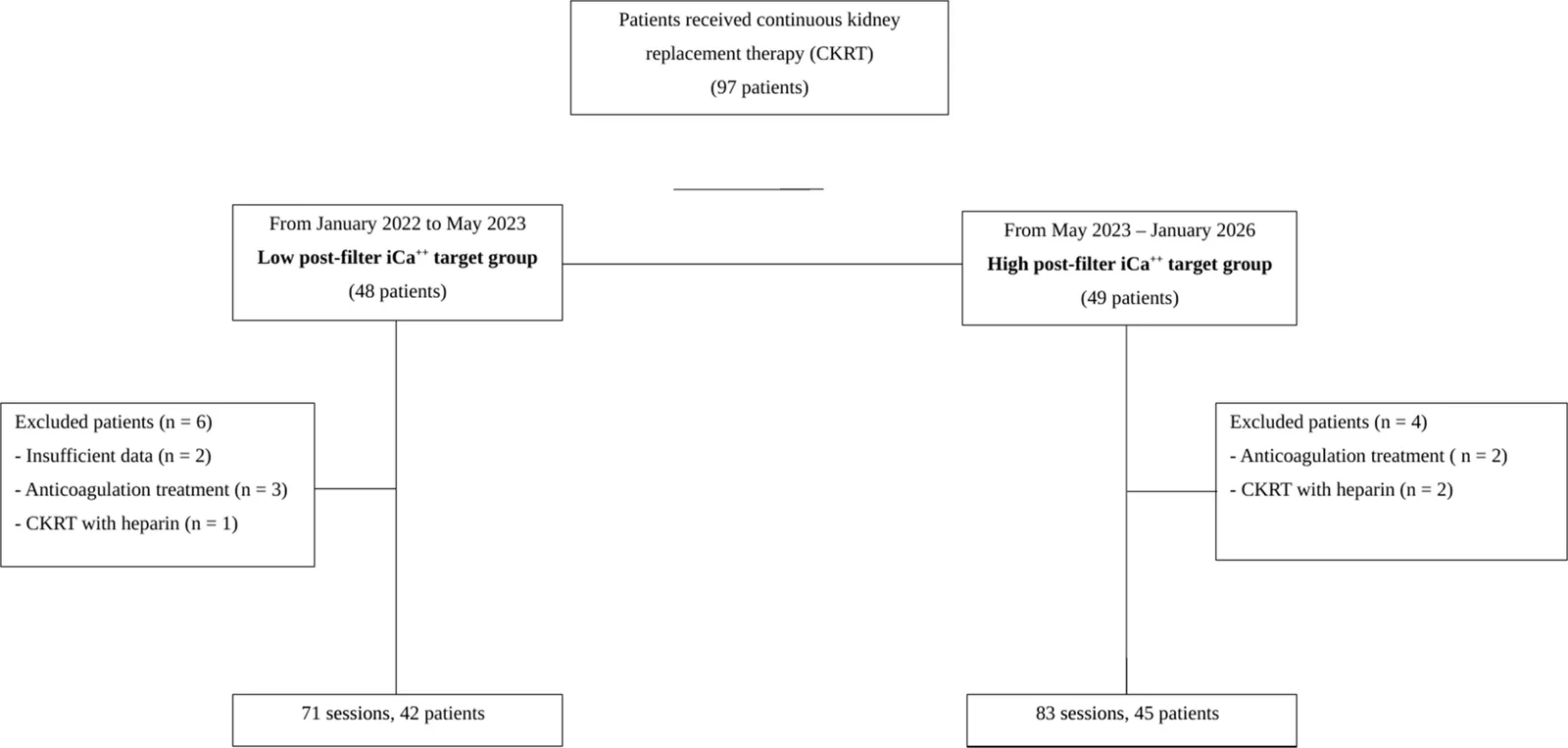
Participant enrollment flowchart. Patient selection flowchart for continuous kidney replacement therapy (CKRT). Patients were stratified into two groups based on post-filter ionized calcium (iCa^2^⁺) targets: low target group (*n* = 48 patients, 71 seasons) enrolled from January 2022 to May 2023, and high target group (*n* = 49 patients, 83 seasons) enrolled from May 2023 to January 2026. Exclusion criteria included insufficient data (*n* = 2), anticoagulation treatment (*n* = 3), and CKRT with heparin (*n* = 1) in the low target group, and anticoagulation treatment (*n* = 2) and CKRT with heparin (*n* = 2) in the high target group

Demographic characteristics, laboratory findings, and CKRT-related parameters were collected from health records. At PICU admission, patient age, weight, sex, comorbidities, and Pediatric Risk of Mortality III (PRISM III) were recorded. CKRT initiation indications included acute kidney injury, electrolyte or acid–base imbalance, fluid overload, hyperammonemia, acute metabolic decompensation, rhabdomyolysis, and tumor lysis syndrome, as determined from physician documentation. Patients discharged alive from PICU were classified as survivors. Prior to CKRT initiation, laboratory data including hemoglobin, platelet count, blood gas parameters (pH, lactate, systemic and pf-iCa^++^), creatinine, urea, and serum electrolytes (sodium, potassium, phosphorus, magnesium, and calcium) were recorded. Serial biochemical measurements and systemic and pf-iCa^++^ blood gas analyses were obtained at protocol-defined intervals throughout CKRT.

### CKRT procedure and citrate anticoagulation

All CKRT treatments were performed using continuous veno-venous hemodiafiltration (CVVHDF) modality with Prismaflex machines (hemofilters HF20, M60, and M100; Gambro, Sweden/Baxter). Commercially available citrate solution (Prismocitrate 18/0) was used as pre-filter anticoagulant fluid. Calcium-free solution (Prismocal B22) was used as dialysate fluid, and calcium-containing solution (Dialisan BG2D) was used as replacement fluid. In all patients, replacement fluid was prescribed and administered as post-filter replacement.

The total clearance rate was generally set at 2–3 L/1.73 m^2^/h, with higher rates up to 5 L/1.73 m^2^/h used for specific indications such as intoxication or hyperammonemia. Citrate was neutralized by continuous calcium infusion using calcium gluconate 10% diluted 1:1 with 5% dextrose, providing a calcium concentration of 116 mmol/L. Calcium was preferably infused through a separate central venous line; when unavailable, it was administered through the circuit return line. The circuit setup is shown in Fig. 2. Subsequent adjustments were made according to post-filter and systemic ionized calcium levels, as detailed in the Online Resource (Supplementary Protocol). The CKRT protocol remained unchanged throughout the study period, except for the target pf-iCa^++^ range.

**Fig. 2:**
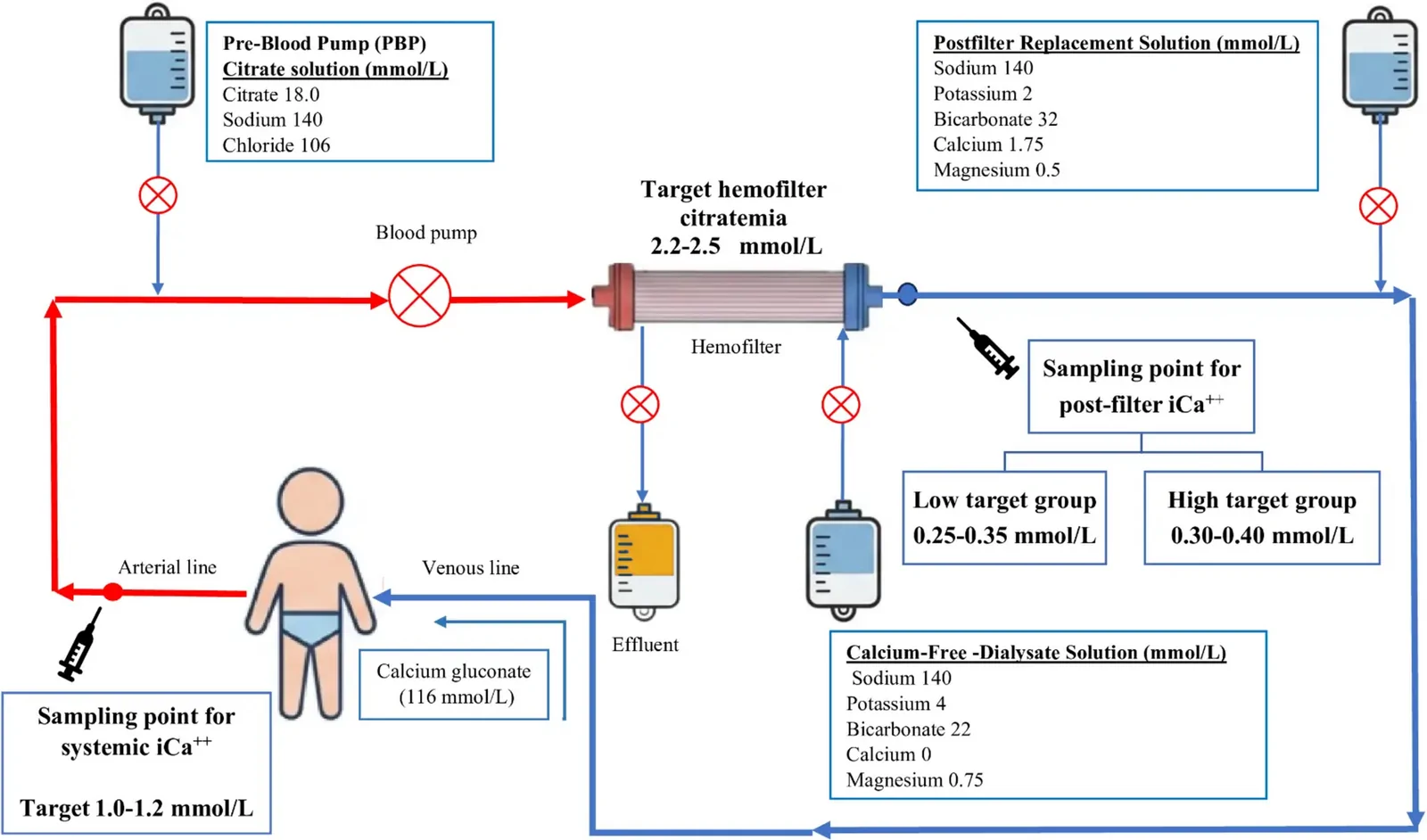
CKRT circuit and citrate protocol schematic. Schematic representation of the continuous kidney replacement therapy (CKRT) circuit with regional citrate anticoagulation protocol. The circuit illustrates pre-blood pump citrate solution composition, target hemofilter citremia (2.2–2.5 mmol/L), post-filter ionized calcium (iCa^++^) sampling points with low target group (0.25–0.35 mmol/L) and high target group (0.30–0.40 mmol/L), systemic iCa^++^target (1.0–1.2 mmol/L), and composition of replacement and dialysate solutions. Red arrows represent arterial line; blue arrows represent venous line and effluent pathway

CKRT data evaluated included treatment duration, filter numbers and usage durations, hemofilter and catheter sizes, CS time (all filters and clotted ones), CKRT prescription (pump flow rate, dialysate flow rate, replacement flow rate), and citrate prescription (citrate infusion dose, citrate flow rate, and citrate load). CKRT and citrate prescriptions were recorded during anticoagulation control and dose adjustments.

CID was defined as the amount of citrate added per liter of blood through the hemofilter (mmol/L). Initial CID was the citrate dose used at CKRT start and agreed with 2.2 or 2.5 mmol/L based on body weight. The citrate solution flow rate was automatically calculated by Prismaflex software when CID and pump flow rate were entered. The average citrate exposure was expressed as “citrate load” in mmol/kg/hours (mmol/kg/h), independently of CID. Citrate load calculated according to the formula [7, 11]:

$$Citrate\;load\;(mmol/kg/h)=Average\;CID\;(mmol/L)\times\frac{Pump\;flow\;rate\;(mL/min)\;\times\;0.06}{Patient\;weight\;(kg)}$$

CS was measured from CKRT initiation with a given filter until filter removal or session termination. CS for clotting circuit was measured from CKRT initiation until filter removal due to clotting. Reasons for termination included circuit clotting, vascular catheter malfunction, scheduled filter replacement after 72 h, end of CKRT treatments (achievement of treatment goals or patient death), and technical issues/alarms. We defined a circuit clotted if transmembrane pressure exceeded 250 mmHg or if visible clots were detected. Catheter malfunction was identified when access pressures remained abnormal—either below −250 mmHg or above + 300 mmHg—indicating mechanical obstruction. CKRT hemofilter manufacturers do not recommend filter use beyond 72 h. However, since this limit was exceeded in previous studies without major complications [26, 27], this threshold was not strictly applied. Some circuits were used longer than 72 h based on clinical assessments and predicted CKRT resolution. For circuits exceeding 72 h, CS and RCA-related complication analyses were limited to the first 72 h.

Since citrate has a short half-life and its anticoagulant effect remains confined to the extracorporeal circuit, we analyzed each circuit as an independent item. All analyses were performed according to the target pf-iCa^++^ group. Patient-level analyses were conducted for baseline demographic and clinical characteristics, whereas circuit-level analyses were used for CKRT prescription parameters, circuit characteristics, citrate system performance, and citrate-related metabolic and electrolyte complications. In the Prismaflex system, the hemofilter is integrated into the circuit; therefore, filter life and circuit life were considered synonymous.

### Statistical analysis

Continuous variables are reported as medians with interquartile ranges (IQR), and categorical variables are expressed as counts, proportions, and percentages. Comparative analyses between groups were conducted using the chi-square test or Fisher’s exact test for categorical data and the Mann–Whitney *U* test for continuous variables. Cox proportional hazards regression analysis was performed to evaluate the effect of the pf-iCa^++^ target group on the CS. The model was adjusted for previously reported risk factors. Hazard ratios (HR) with 95% confidence intervals (CI) were calculated [4, 28–30].

For the primary outcome of CS, power analysis was performed with *α* = 0.05 (two-tailed), statistical power of 0.80, and an expected medium effect size (Cohen’s *d* = 0.5). Using G*Power 3.1 software, a minimum sample size of 64 circuits per group (*n* = 128 total) was determined to be required to detect a clinically meaningful difference in CS. For binary outcomes, including citrate-related clinically relevant metabolic disturbances (hypocalcemia and T/iCa^++^ ratio > 2.5), generalized estimating equations (GEE) with a binomial distribution and logit link function were used, assuming an exchangeable correlation structure. Longitudinal changes in citrate dose and citrate load were analyzed using linear mixed models (LMM), with patients specified as a random effect to account for inter-individual variability.

For both GEE and LMM analyses, the study group, time, and group × time were included as fixed effects. Time was modeled as a categorical variable corresponding to predefined 4-h intervals. Model and covariance structure selection was primarily guided by the lowest Akaike’s information criterion (AIC) while also considering model fit, stability, and clinical interpretability. Pairwise comparisons between groups at each time point were performed using estimated marginal means with Bonferroni adjustment for multiple comparisons (corrected *α* = 0.0026). Figures were generated based on estimated marginal means, with corresponding pairwise comparison p-values indicated below the *x*-axis.

A two-sided *p*-value of < 0.05 was considered statistically significant. Statistical analyses were conducted using IBM SPSS Statistics for Windows, version 25.0 (IBM Corp., Armonk, NY, USA).

## Results

During four years, 1620 children were admitted to our PICU, of whom 97 required CKRT. After excluding ten who did not meet criteria, 87 patients remained for analysis (Fig. 1). Of these, 42 patients (71 circuits) were classified under LT-pf-iCa^++^ strategy, and 45 patients (83 circuits) under HT-pf-iCa^++^ strategy. Baseline characteristics were comparable between groups (Table 1), except for CKRT indications. Rhabdomyolysis was more frequent in the LT group (19% vs. 0.0%, *p* = 0.02).

**Table 1:** Baseline characteristics and clinical outcomes according to post-filter ionized calcium target groups

Variable	All patients^a^ (*n* = 87)	Low-target pf-iCa^++ a^ (*n* = 42)	High-target pf-iCa^++ a^ (*n* = 45)	*p*
Age (years), median (IQR)	5.90 (3.70–8.55)	6.45 (3.62–8.17)	5.60 (3.80–8.60)	0.9
Sex (Male), *n* (%)	40 (46.0%)	19 (45.2%)	21 (46.7%)	0.9
Weight (kg), median (IQR)	18.50 (13.90–28.40)	18.50 (13.27–29.15)	18.60 (15.30–28.40)	0.9
Weight < 10 kg, *n* (%)	10 (11.5%)	6 (14.3%)	4 (8.9%)	0.51
PRISM III score, median (IQR)	13 (9–20)	14 (9–21)	12 (8–19)	0.40
Comorbidities, *n *(%)	32 (36.8%)	15 (35.7%)	17 (37.8%)	0.9
CKRT indication, *n* (%)				
Acute kidney injury	61 (70.1%)	30 (71.4%)	31 (68.9%)	0.9
Electrolyte/acid–base disturbance	22 (25.3%)	7 (16.7%)	15 (33.3%)	0.1
Fluid overload	19 (21.8%)	10 (23.8%)	9 (20.0%)	0.87
Rhabdomyolysis	8 (9.2%)	8 (19.0%)	0 (0.0%)	**0.02**
Acute attack of metabolic disease	3 (3.4%)	1 (2.4%)	2 (4.4%)	0.9
Tumor lysis syndrome	3 (3.4%)	1 (2.4%)	2 (4.4%)	0.9
Hyperammonemia	2 (2.3%)	1 (2.4%)	1 (2.2%)	0.9
Vascular access site, *n* (%)				
Internal jugular	73 (83.9%)	35 (83.3%)	38 (84.4%)	0.80
Subclavian	3 (3.4%)	2 (4.8%)	1 (2.2%)	
Femoral	11 (12.6%)	5 (11.9%)	6 (13.3%)	
Laboratory values at CKRT initiation, median (IQR)				
Hemoglobin (g/dL)	10.9 (9.8–11.8)	10.9 (10.3–11.5)	10.8 (9.7–11.8)	0.58
Platelet (10^3^/µL)	148 (114–171)	154 (108–176)	144 (122–165)	0.71
Creatinine (mg/dL)	3.48 (1.89–6.09)	2.67 (1.75–5.53)	3.96 (2.56–6.54)	0.10
Urea (mg/dL)	114.00 (74.70–179.10)	116.20 (65.55–176.62)	110.10 (81.80–178.00)	0.9
pH	7.22 (7.14–7.29)	7.24 (7.14–7.31)	7.21 (7.14–7.28)	0.72
Lactate (mmol/L)	2.80 (2.15–3.70)	2.90 (2.25–3.72)	2.70 (2.10–3.60)	0.75
Potassium (mmol/L)	3.97 (3.31–5.14)	3.76 (3.31–4.83)	4.02 (3.38–5.68)	0.49
Sodium (mmol/L)	135.40 (131.70–139.55)	135.15 (132.45–140.60)	135.80 (131.10–138.80)	0.33
Phosphorus (mg/dL)	6.10 (4.45–8.90)	5.90 (4.50–9.75)	6.10 (4.50–8.20)	0.9
Magnesium (mg/dL)	1.90 (1.50–2.50)	1.90 (1.35–2.60)	1.90 (1.60–2.30)	0.9
Total calcium (mg/dL)	8.0 (6.7–9.6)	8.1 (6.9–10.1)	7.8 (6.4–9.4)	0.43
Systemic ionized calcium (mmol/L)	1.06 (1.00–1.12)	1.06 (0.99–1.14)	1.07 (1.02–1.12)	0.39
PICU length of stay (days), median (IQR)	9 (6–13)	8 (6–12)	9 (6–13)	0.79
PICU mortality, *n* (%)	17 (19.5%)	10 (23.8%)	7 (15.6%)	0.48

*IQR*, interquartile range; *PRISM III*, Pediatric Risk of Mortality III score; *CKRT*, continuous kidney replacement therapy; *PICU*, pediatric intensive care unit

^a^Data are presented at the patient level as number of patients. Total patients = 87 (low target, 42; high target, 45)

### CKRT parameters and circuit survival

A total of 8433 h of CKRT were delivered using 154 hemofilters (LT-pf-iCa^++^ 3827 h, 71 filters; HT-pf-iCa^++^ 4606 h, 83 filters) (Table 2). Median CS did not differ between groups, either overall (49 vs. 48 h, *p* = 0.76) or among circuits terminated due to clotting (41 vs. 40 h, *p *= 0.70). Dialysis prescription parameters were comparable between groups; however, weight-adjusted pump flow rate was higher in the HT-pf-iCa^++^ group (4.8 vs. 5.1 mL/kg/min, *p* < 0.001). Median citrate dose and citrate load were higher in the LT-pf-iCa^++^ group (respectively; 2.9 vs. 2.6 mmol/L, *p* < 0.001; 0.76 vs. 0.72 mmol/kg/h, *p* = 0.03). The proportion of pf-iCa^++^ values within the target range and filtration fraction (FF%) did not differ between groups. Hemofilter clotting rates were similar (21.1% vs. 24.1%, *p* = 0.70), as were other causes of circuit termination (Table 2).

**Table 2:** Continuous kidney replacement therapy (CKRT) parameters and circuit characteristics according to post-filter ionized calcium target groups

Variable	All^a^ (*n* = 154)	Low-target pf-iCa^++ a^ (*n* = 71)	High-target pf-iCa^++ a^ (*n* = 83)	*p*
Total CKRT duration (h)	8433	3827	4606	
CKRT duration per patient (h), median (IQR)	94 (68–122)	83 (62–115)	104 (74–124)	0.20
Hemofilters per patient, median (IQR)	2.00 (1.50–2.00)	2.00 (1.00–2.00)	2.00 (2.00–2.00)	0.15
Circuit survival (h), median (IQR)	49 (37–69)	49 (36–66)	48 (38–71)	0.76
Circuit survival for clotting hemofilter (h), median (IQR)	40 (33–48)	41 (35–49)	40 (31–48)	0.70
Filter surface area (m^2^), *n* (%)				
0.2	17 (11.0%)	9 (12.7%)	8 (9.6%)	0.73
0.6	79 (51.3%)	31 (43.7%)	48 (57.8%)	0.11
0.9	58 (37.7%)	31 (43.7%)	27 (32.5%)	0.21
Pump flow rate, median (IQR)				
mL/min	96.5 (79.0–126.0)	97.0 (79.0–121.0)	96.0 (79.0–132.0)	0.23
mL/kg/min	4.9 (3.7–5.8)	4.8 (3.6–5.6)	5.1 (3.9–6.0)	** < 0.001**
Dialysate flow rate, median (IQR)				
mL/h	543.0 (343.0–947.8)	543.0 (356.0–1008.5)	553.0 (341.5–925.5)	0.9
mL/kg/h	26.7 (19.9–32.0)	26.3 (19.8–31.5)	26.8 (20.1–33.0)	0.25
mL/1.73 m^2^/h	1214.1 (811.0–1536.6)	1188.2 (805.6–1477.0)	1308.1 (811.0–1573.1)	0.36
Post-filter replacement flow rate, median (IQR)				
mL/h	439.0 (247.8–657.8)	436.0 (247.0–691.0)	442.0 (270.0–644.0)	0.73
mL/kg/h	20.3 (16.2–23.7)	19.6 (16.2–23.5)	20.4 (16.3–23.8)	0.89
mL/1.73 m^2^/h	929.8 (663.8–1136.5)	920.0 (660.2–1130.5)	972.8 (664.6–1137.4)	0.87
Citrate administration parameters, median (IQR)				
Citrate infusion dose (mmol/L)	2.7 (2.5–3.0)	2.9 (2.6–3.2)	2.6 (2.5–2.7)	** < 0.001**
Citrate flow rate (mL/h)	395.4 (328.9–520.3)	407.9 (341.4–518.2)	391.3 (328.9–547.4)	0.9
Citrate flow rate (mL/1.73 m^2^/h)	872.0 (763.5–1014.7)	862.7 (737.9–972.4)	872.0 (786.9–1041.1)	0.41
Citrate load (mmol/kg/h)	0.75 (0.61–0.84)	0.76 (0.63–0.87)	0.72 (0.58–0.81)	**0.03**
Post-filter iCa^++^ within target range (%), median (IQR)	84.6 (78.3–90.9)	83.3 (77.8–90.5)	85.0 (79.1–91.3)	0.31
Filtration fraction (%), median (IQR)	23 (19–27.5)	23 (19–28)	23 (20–27)	0.83
Dialysis catheter size (French), *n* (%)				
7	5 (3.2%)	3 (4.2%)	2 (2.4%)	0.66
8	19 (12.3%)	8 (11.3%)	11 (13.3%)	0.89
9	54 (35.1%)	25 (35.2%)	29 (34.9%)	0.9
10	56 (36.4%)	26 (36.6%)	30 (36.1%)	0.9
11	9 (5.8%)	5 (7.0%)	4 (4.8%)	0.73
12	11 (7.1%)	4 (5.6%)	7 (8.4%)	0.55
Reasons for circuit discontinuation, *n* (%)				
Filter clotting	35 (22.7%)	15 (21.1%)	20 (24.1%)	0.70
Catheter dysfunction	18 (11.7%)	8 (11.3%)	10 (12.0%)	0.9
Scheduled filter replacement after 72 h	27 (17.5%)	15 (21.1%)	12 (14.5%)	0.30
End of CKRT	65 (42.2%)	29 (40.8%)	36 (43.4%)	0.87
Technical issues/alarms	9 (5.8%)	4 (5.6%)	5 (6.0%)	0.9

*CKRT*, continuous kidney replacement therapy; *IQR*, interquartile range; *h*, hour; *m*^*2*^, square meter; *mL/min*, milliliters per minute; *mL/kg/min*, milliliters per kilogram per minute; *mL/h*, milliliters per hour; *mL/1.73 m*^*2*^*/h*, milliliters per 1.73 square meters per hour

^a^Data are presented at the filter level as number of hemofilters. A total of 154 hemofilters were included (Low-target pf-iCa + +, 71; high-target pf-iCa + +, 83)

Cox proportional hazards regression identified factors associated with circuit clotting (Table 3). FF% was independently associated with increased hemofilter clotting risk (HR 1.086 per 1% increase; 95% CI 1.019–1.157, *p* = 0.011). The target pf-iCa^++^ strategy showed no significant difference between HT and LT pf-iCa^++^ groups (HR 1.028; 95% CI 0.501–2.100, *p* = 0.944).

**Table 3:** Cox proportional hazards regression analysis of risk factors for circuit clotting

Variable	HR	SE	95% CI	*p*
Hemoglobin (g/dL)	1.051	0.186	0.727–1.513	0.806
Platelet (10^4^/µL)	0.963	0.058	0.860–1.080	0.515
Vascular access (femoral vein)	1.003	0.005	0.992–1.014	0.574
Filter size 0.2 m^2^	1.030	0.136	0.789–1.340	0.830
Pump flow rate (mL/kg/min)	1.176	0.113	0.941–1.469	0.153
Group (high target pf-iCa^++^)	1.028	0.366	0.501–2.100	0.944
Citrate load (per 0.1 mmol/kg/h)	0.679	0.260	0.408–1.130	0.137
Filtration fraction (%)	1.086	0.032	1.019–1.157	**0.011**

*HR*, hazard ratio; *CI*, confidence interval; *g/dL*, grams per deciliter; *µL*, microliter; *mL/kg/h*, milliliters per kilogram per hour; *mL/kg/min*, milliliters per kilogram per minute; *m*^*2*^, square meter

### Metabolic and electrolyte disturbance and citrate administration

In the LT-pf-iCa^++^ group, the incidence of hypocalcemia (17.5% vs. 12.9%, *p* = 0.04), metabolic alkalosis (31.9% vs. 22.8%, *p* < 0.001), and a T/iCa^++^ ratio > 2.5 (24.5% vs. 15.4%, *p* < 0.001) was significantly higher compared to the HT-pf-iCa^++^ group. No statistically significant differences were observed between the groups concerning other measured metabolic abnormalities (Supplementary Table 1). The temporal and group-based longitudinal analyses of key citrate-related parameters and metabolic disturbances (citrate dose, citrate load, hypocalcemia, and T/iCa^++^ ratio > 2.5) are summarized in Table 4 and illustrated in Figs. 3 and 4.

**Table 4:** Model-based longitudinal regression analyses by post-filter ionized calcium target groups

Variables^a^	Low Target pf-iCa^++ b^ (*n* = 907)	High Target pf-iCa^++ b^ (*n* = 1133)	Group effect		Time effect		Group × time effect		Effect size^e^ (Cohen’s h/d)
			**Effect estimate (OR or β, 95% CI)**	***p***	**Effect estimate (OR or β, 95% CI)**	***p***	**Effect estimate (OR or β, 95% CI)**	***p***	
**Continuous variables**^**c**^	**Mean ± SD**								
Citrate dose (mmol/L)	2.901 ± 0.307	2.601 ± 0.165	0.281 (0.236–0.327)	** < 0.001**	0.010 (0.010–0.010)	** < 0.001**	0.008 (0.007–0.008)	** < 0.001**	1.259
Citrate load (mmol/kg/h)	0.876 ± 0.264	0.722 ± 0.248	0.159 (0.082–0.237)	** < 0.001**	0.003 (0.002–0.003)	** < 0.001**	0.002 (0.002–0.002)	** < 0.001**	0.603
**Binary variables**^**d**^	***n* (%)**								
Hypocalcemia	159 (17.5%)	146 (12.9%)	1.472 (1.090–1.988)	**0.01**	1.014 (1.007–1.022)	** < 0.001**	1.014 (1.003–1.025)	**0.01**	0.130
T/iCa^++^ Ratio > 2.5	222 (24.5%)	175 (15.4%)	1.925 (1.537–2.412)	** < 0.001**	1.025 (1.018–1.033)	** < 0.001**	1.013 (1.002–1.023)	**0.02**	0.227

*OR*, odds ratio; *CI*, confidence interval; *iCa*, ionized calcium; *SD*, standard deviation

^a^All models included study group, time, and group × time as fixed effects. Time was modeled as a categorical variable corresponding to predefined 4-h measurement intervals. Pairwise comparisons between groups at each time point were performed using estimated marginal means. Bonferroni correction was applied for multiple comparisons. The high target pf-iCa^++^ group was used as the reference category for group comparisons

^b^Data are presented as number of measurements. Total measurements = 2040 (low target, 907; high target, 1133)

^c^Continuous outcomes (citrate dose and citrate load) were analyzed using linear mixed models (LMM). Effect estimates are presented as regression coefficients (β) with 95% confidence intervals Positive β coefficients indicate higher values over time, whereas negative values indicate lower values. To facilitate clinical interpretation, changes over a 24-h period were approximated by scaling the estimated time coefficients accordingly

^d^Binary outcomes (hypocalcemia, metabolic acidemia, metabolic alkalemia, and T/iCa^++^ ratio > 2.5) were analyzed using generalized estimating equations (GEE) with a binomial distribution and logit link function, assuming an exchangeable correlation structure. Effect estimates are presented as odds ratios (OR) with 95% confidence intervals (CI)

^e^Effect size (Cohen’s h for binary outcomes and Cohen’s d for continuous outcomes) was calculated to facilitate clinical interpretation

**Fig. 3:**
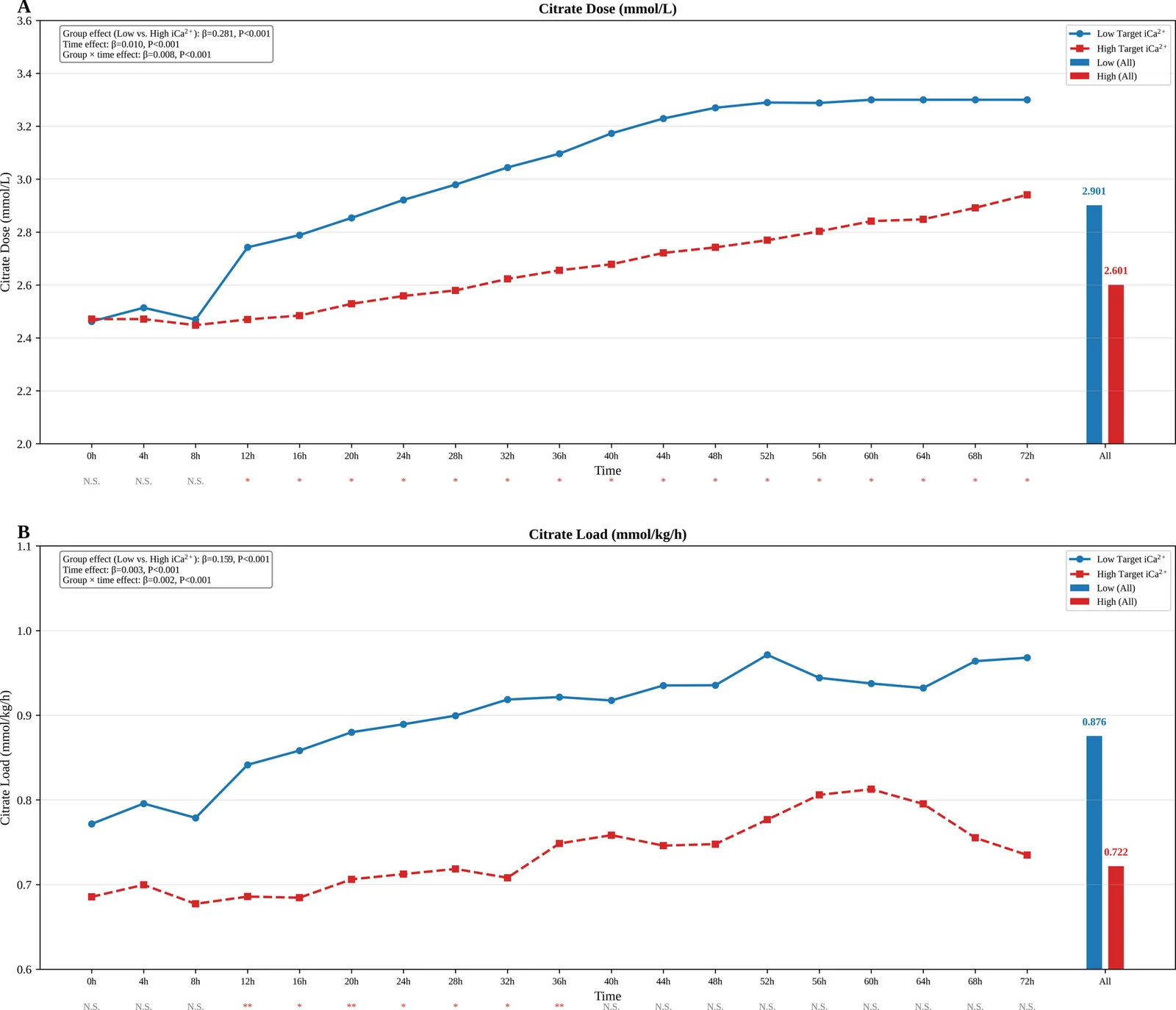
Citrate dose and citrate load over time. Longitudinal changes in citrate parameters during the first 72 h after CKRT initiation. **a** Citrate Dose (mmol/L) **b** Citrate Load (mmol/kg/h). Results are represented as mean values along with their 95% confidence intervals displaying the predicted marginal means each point calculated from generalized estimating equations (GEE) analysis. Statistically significant differences in changes from baseline (CKRT initiation) effects of group, time, and group × time are indicated by *p < 0.001, **p < 0.002; NS, non-significant; β, beta coefficient; CKRT, continuous kidney replacement treatment

**Fig. 4:**
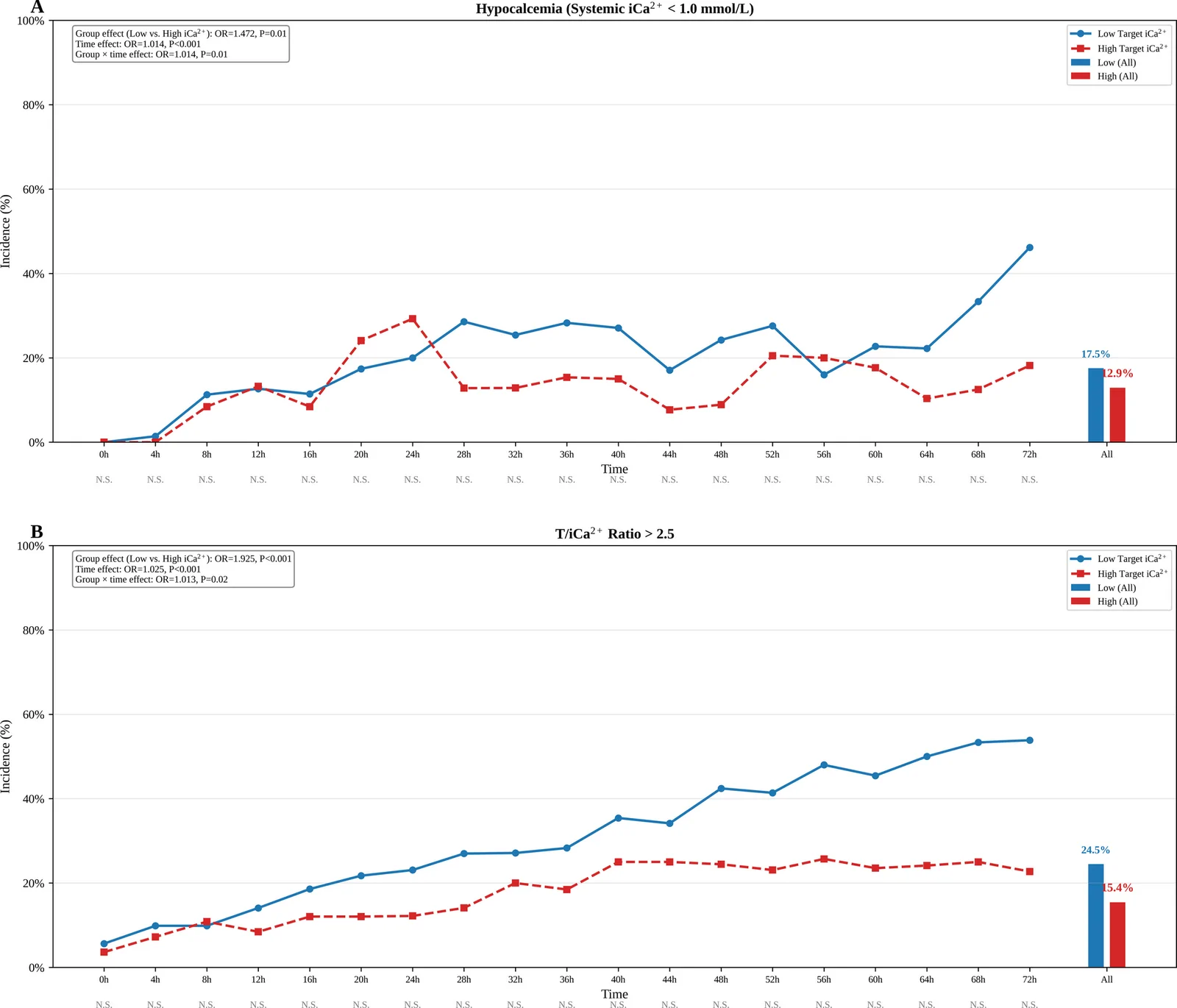
Hypocalcemia incidence and T/iCa^++^ ratio over time. Longitudinal analysis of citrate-related complications during the first 72 h after CKRT initiation. **a** Hypocalcemia (systemic iCa^++^ < 1.0 mmol/L). **b** T/iCa^++^ ratio > 2.5. Results are represented as mean values along with their 95% confidence intervals displaying the predicted marginal means each point calculated from generalized estimating equations (GEE) analysis. N.S., non-significant; OR, odds ratio; CKRT, continuous kidney replacement treatment

### Citrate dose (mmol/L)

The LMM analysis revealed a significant group effect: the LT-pf-iCa^++^ group required on average 0.28 mmol/L higher citrate dose than the HT-pf-iCa^++^ group (*β* = 0.281, 95% CI 0.236–0.327, *p* < 0.001). Citrate dose increased by 0.010 mmol/L per 24 h in the entire cohort. (*β* 0.010, *p* < 0.001), with an additional 0.008 mmol/L per 24 h increase in the LT group (*β* 0.008, *p* < 0.001). The mean citrate dose over time is shown in Fig. 4a. Pairwise comparisons with Bonferroni correction demonstrated that the citrate dose was significantly higher in the LT-pf-iCa^++^ group from 12 h through 72 h (all *p* < 0.001).

### Citrate load (mmol/kg/h)

A significant group effect was found: the LT-pf-iCa^++^ group had on average 0.16 mmol/kg/h higher citrate load than the HT-pf-iCa^++^ group (*β* = 0.159, 95% CI 0.082–0.237, *p* < 0.001). Citrate load increased by 0.003 mmol/kg/h per 24 h in the entire cohort (*β* = 0.003 per 24 h, 95% CI 0.002–0.003, *p* < 0.001), with an additional 0.002 mmol/kg/h per 24 h increase in the LT group (*β* 0.002, *p* < 0.001). The mean citrate load over time is presented in Fig. 4b. Pairwise comparisons with Bonferroni correction showed that the citrate load was significantly higher in the LT-pf-iCa^++^ group from 12 h through 48 h (*p* < 0.001 to *p* = 0.002), with no significant differences observed thereafter.

### Hypocalcemia (Systemic iCa < 1.0 mmol/L)

GEE analysis demonstrated 47% higher odds of hypocalcemia in the LT-pf-iCa^++^ group compared with the HT group (OR 1.47, 95% CI 1.09–1.99, *p* = 0.01). Hypocalcemia risk increased by 1.4% per 4-h interval in the entire cohort (OR 1.014, *p* < 0.001), with an additional 1.4% increase per interval in the LT group, as indicated by a significant group × time effect (OR 1.014, *p* = 0.01). The incidence of hypocalcemia over time is illustrated in Fig. 3a.

### T/iCa^++^ ratio > 2.5

The LT-pf-iCa^++^ group had 93% higher odds of a T/iCa^++^ ratio > 2.5 compared with the HT group (OR 1.93, 95% CI 1.54–2.41, *p* < 0.001). The odds increased by 2.5% per 4-h interval over time in the entire cohort (OR 1.025, *p* < 0.001), with an additional 1.3% increase per interval in the LT group, as indicated by a significant group × time effect (OR 1.013, *p* = 0.02). The incidence of T/iCa^++^ ratio > 2.5 over time is depicted in Fig. 3b. Despite the significant overall group effect, pairwise comparisons with Bonferroni correction showed no statistically significant differences between groups at any individual time point.

## Discussion

This study demonstrates that, in pediatric patients undergoing CKRT with a low initial CID protocol (2.2–2.5 mmol/L), increasing the target pf-iCa^++^ range from 0.25–0.35 mmol/L to 0.30–0.40 mmol/L reduces both citrate dose and cumulative citrate load without shortening CS until clotting. FF% was the only variable independently associated with an increased risk of hemofilter clotting. The LT-pf-iCa^++^ protocol was associated with a 1.93-fold higher risk of CA, as well as significantly increased risks of systemic hypocalcemia and metabolic alkalosis. Together, these results position pf-iCa^++^ targeting as a central modulator of citrate exposure and metabolic safety rather than a simple surrogate of anticoagulation intensity.

Pediatric CKRT is technically more challenging than adult CKRT due to the use of smaller-diameter catheters and hemofilters with lower surface area, as well as lower prescribed pump flow rates. These factors place pediatric patients at an inherent disadvantage in terms of CS [30]. Consequently, effective anticoagulation is essential to ensure uninterrupted CKRT delivery, particularly in children [30, 31]. In recent years RCA has gained prominence in both adult and pediatric CKRT owing to its association with prolonged CS and reduced bleeding risk [4, 5, 8–10, 30, 32]. However, no consensus exists regarding the optimal initial CID in protocols, with reported starting doses ranging widely from 2.0 to 5.0 mmol/L [4, 11, 19, 32–36].

Citrate achieves anticoagulation by chelating iCa^++^, an essential cofactor in the coagulation cascade [37]. In RCA, pf-iCa^++^ is the only surrogate marker used to monitor anticoagulation efficacy [16]. However, no consensus exists regarding the optimal pf-iCa^++^ target. Current recommendations are based primarily on underpowered studies [17, 18], and reported targets range from 0.25 to 0.50 mmol/L [11, 16, 28, 38], with most protocols aiming for values < 0.35 mmol/L [4, 11, 21–24, 39]. Citrate dosing during CKRT is dynamically titrated according to the target pf-iCa^++^ level. Therefore, the pf-iCa^++^ target is the principal determinant of citrate exposure. Targeting unnecessarily low pf-iCa^++^ levels may not confer additional anticoagulation benefit, yet may increase citrate exposure and its associated metabolic complications. Although recent years have seen increasing research on citrate dosing strategies, a limited number of studies have specifically evaluated the impact of pf-iCa^++^ target ranges, the key surrogate of anticoagulation, on both CS- and citrate-related adverse metabolic effects and these studies have been conducted exclusively in adult populations [21, 25].

Consistent with previously reported pediatric populations, the most common CKRT indications in our cohort, in descending order of frequency, were acute kidney injury, electrolyte and acid–base disturbances, and fluid overload [1, 2]. Except for rhabdomyolysis, the distribution of CKRT indications was similar between groups. Rhabdomyolysis was observed exclusively in the low-target pf-iCa^+^^+^group (19.0% vs. 0.0%), reflecting the temporal clustering of earthquake-related crush syndrome admissions from the Kahramanmaraş earthquake (February 6, 2023) during the low-target treatment phase.

At our center, a low initial CID protocol has been adopted over the past 4 years with the aim of minimizing cumulative citrate exposure. Furthermore, to attenuate the time-dependent escalation in citrate dose observed during prolonged CKRT, the protocol was revised after May 2023 by increasing the target pf-iCa^++^ range from 0.25–0.35 mmol/L to 0.30–0.40 mmol/L. Importantly, increasing the pf-iCa^++^ target did not result in a higher risk of hemofilter clotting, with comparable CS between groups (41 h vs. 40 h). Although CS in our cohort was administratively capped at 72 h, the observed median lifespan was within the range reported in previous pediatric studies applying different initial CID strategies (34.0–71.3 h) [7, 11, 26, 27, 40]. To our knowledge, only one study has investigated the effect of different pf-iCa^++^ target ranges within the same initial CID protocol on CS, and it was performed in adult patients with CKRT. This study, similar to our own research, demonstrated that increasing the pf-iCa^++^ target from 0.25–0.35 mmol/L to 0.30–0.40 mmol/L did not lead to a reduction in CS until clotting occurred [21].

In patients receiving RCA-CKRT, the citrate load is primarily influenced by CID and the pump flow rate. While CID remains unchanged, an increase in the pump flow rate results in a higher citrate load. In pediatric patients, pump flow rates are prescribed in mL/kg/min, resulting in higher flow rates compared to adults. As citrate infusion rates are directly correlated with pump flow, children are consequently exposed to substantially higher citrate loads on a mmol/kg basis [41, 42]. Consequently, optimization of pump flow rates represents one strategy to reduce citrate load and associated complications in pediatric patients [11, 21, 42].

Persic et al. demonstrated that maintaining pump flow rate below 9 mL/kg/min in neonates and infants prevents citrate-related complications [7]. Yessayan et al. showed that maintaining a constant ratio of citrate flow rate to pump flow rate prevents CA and significantly reduces citrate-related complications [43]. Additionally, using lower CID (< 3 mmol/L) and targeting higher pf-iCa^++^ levels (> 0.5 mmol/L) represent alternative strategies to prevent citrate-related complications [11, 21, 42]. In our cohort, targeting a higher pf-iCa^++^ level to reduce citrate exposure did not compromise anticoagulation efficacy. Despite higher pump flow rates in the HT pf-iCa^++^ group (5.1 vs. 4.8 mL/kg/min), this strategy was associated with lower CID requirement (2.6 vs. 2.9 mmol/L), lower citrate load (0.72 vs. 0.76 mmol/kg/h), and reduced risk of citrate-related metabolic and electrolyte disturbances, including CA, hypocalcemia, and metabolic alkalosis, consistent with previous reports [4, 19, 32].

Limited retrospective adult studies have assessed the impact of modifying pf-iCa^++^ targets on citrate exposure and citrate-related metabolic disturbances during CKRT [21, 25]. Assefi et al. analyzed data from 1037 CKRT sessions performed in 279 critically ill patients and reported that increasing the pf-iCa^++^ target from 0.25–0.35 to 0.30–0.40 mmol/L resulted in reduced daily median citrate exposure (from 180 to 118 mmol/day) without significantly decreasing metabolic complications [21]. In a second study of 240 CKRT sessions in 40 patients undergoing cardiac surgery, higher pf-iCa^++^ targets (< 0.50 vs. < 0.40 mmol/L) were compared; however, no difference was found in CS or citrate exposure [25]. Protocol variations limit adequate evaluation of pf-iCa^++^ target effects on metabolic disturbances. Pediatric CKRT differs from adult practice in weight-adjusted pump flow rates and citrate exposure per kilogram, rendering adult data extrapolation potentially misleading. Our retrospective cohort study is the first to evaluate this question in pediatric CKRT, addressing a gap in the pediatric literature.

Systemic hypocalcemia occurs in up to 60% of pediatric patients receiving RCA during CKRT [4, 8, 32, 42–44]. In our cohort, the overall incidence was 15.7%. Longitudinal analyses revealed that hypocalcemia risk increased over time, with a notably more pronounced temporal rise in the LT-pf-iCa^++^ group (17.5% vs. 12.9%, *p* = 0.04). This pattern reflects the progressive increase in citrate dose required to maintain lower pf-iCa^++^ targets, enhancing systemic citrate–calcium complex formation and reducing circulating ionized calcium levels. Poh et al. demonstrated that in adult CKRT patients, higher initial CID (3.0 mmol/L) versus 2.5 mmol/L resulted in increased hypocalcemia incidence at the same pf-iCa^++^ target; however, this difference resolved after 12 h when citrate doses converged [28]. These findings suggest that hypocalcemia risk is primarily associated with the temporal progression of citrate dosing and cumulative citrate exposure rather than initial dosing. Age-related differences in citrate metabolism and protocol variations, such as calcium replacement rates, may contribute to this observation.

The most feared complication of RCA is CA [7]. T/iCa^++^ ratio > 2.5 is commonly used as an indirect marker of CA [45]. No universally accepted definition of CA exists in pediatric or adult populations. Studies define CA either based on T/iCa^++^ ratio alone, reporting higher incidences, or using composite criteria with acid–base status and anion gap, showing lower rates [4, 7, 40]. Despite the absence of consensus, the reported incidence of CA in pediatric CKRT studies ranges up to 39.3% [4, 7, 11, 34, 40]. In our cohort, the incidence was 19.5% and significantly higher in the LT-pf-iCa^++^ group (24.5% vs. 15.4%), with increased risk over time, particularly in the LT group. In contrast, adult studies have reported that 70–90% of CA events occur within the first 24 h [45–47], with prolonged treatment duration conferring no additional risk [28]. However, in a pediatric series by Kihtir et al., CA risk increased with CKRT duration, peaking in circuits used beyond 72 h (odds ratio 2.1) [11].

To our knowledge, this is the first study comparing different pf-iCa^++^ target ranges in pediatric patients receiving RCA during CKRT [48], with a focus on CS and adverse effects related to citrate [49]. The similarity in prescription and monitoring algorithms between protocols, including initial CID, starting parameters, equipment, and replacement solutions, allowed reliable assessment of pf-iCa^++^ target differences. Data were extracted from well-maintained PICU and CKRT records. CKRT parameters were systematically recorded using a standardized CKRT chart, enabling consistent data collection and minimizing missing data. However, our study has limitations. First, this single-center, retrospective observational study limits causal inference and generalizability. Second, implementing protocols during different periods may have introduced temporal bias in clinical practices. Third, circuit pressures and staff responses to pump alarms were not systematically analyzed, though inadequate troubleshooting can contribute to circuit clotting independent of anticoagulation strategy.

RCA achieves anticoagulation by lowering iCa^++^ levels at the circuit. However, limited evidence regarding optimal pf-iCa^++^ target in RCA-CKRT prompted us to investigate whether a higher pf-iCa^++^ range could safely reduce citrate exposure. In our study, increasing pf-iCa^++^ target did not compromise anticoagulation efficacy, while significantly reducing citrate dose and cumulative citrate load. This reduction translated into a lower risk of citrate-related metabolic complications. These findings suggest that pf-iCa^++^ may be considered within a broader therapeutic range without loss of circuit performance. Such an approach may improve treatment stability, reduce the need for frequent dose adjustments, and potentially decrease workload. Moreover, minimizing citrate exposure may enhance the safety of RCA in patients with relative contraindications such as hepatocellular dysfunction or tissue hypoxia.

## Supplementary Information

Below is the link to the electronic supplementary material.

ESM1 Graphical Abstract (PPTX 558 KB)

ESM2 (DOCX 29.3 KB)

## Declarations

### Disclaimer

This manuscript is an original work not published elsewhere, including text and graphics. Any similarities to other works are coincidental. This manuscript was prepared with AI assistance, specifically using Paperpal for language editing. The final content has been reviewed and approved by the authors to ensure accuracy and originality.
