# Supplementary material for: Impact of post-filter ionized calcium target range on circuit survival and citrate-related complications in pediatric continuous kidney replacement therapy
Source: Pediatr Nephrol. 2026 Jul 8;41(12):4547–59. doi: 10.1007/s00467-026-07440-1 (PMC13672384; doi:10.1007/s00467-026-07440-1)
Supplement: Supplementary file 1 [file 467_2026_7440_MOESM2_ESM.docx]

**Regional citrate anticoagulation protocol for critically ill children requiring continuous kidney replacement therapy**

**Starting Parameters**

a) **Blood flow rate:** 3–8 mL/kg/min (maximum 150 mL/min) with respect to patient weight, catheter size and hemofilter surface [49].

**Table S1.** Blood flow rates according to body weight in CKRT patients

| Patient weight (kg) | Blood flow rate (mL/kg/min) |
| --- | --- |
| 3-6 | 8-12 |
| 6-15 | 5-8 |
| 15-30 | 4-6 |
| >30 | 2-4 |

CKRT: continuous kidney replacement therapy

a) **Total effluent dose:** Between 2–3 L/1.73m^2^/h for the majority of patients, while higher total clearance rates (up to a maximum of 5 L/1.73m^2^/h) were reached in cases with specific indications, such as intoxication or hyperammonemia.
b) **Citrate dose:** Since January 2022, all RCAs were administered with a low initial citrate infusion dose. Lower dosages were administered as 2.2 mmol/L for patients weighing ≤10 kg and as 2.5 mmol/L for those weighing >10 kg. Citrate flow was coupled to blood flow and adjusted by the CRRT device to achieve the prescribed citrate dose (2.2 or 2.5 mmol/L). All CRRTs receiving RCA were performed with pre-filter citrate anticoagulation and post-filter replacement fluid.
c) **Filtration fraction (FF%):** While regulating dialysate and replacement flows, care was taken not to increase the filtration fraction above 25%.
d) **Modality:** Continuous veno-venous hemodiafiltration was administered to all subjects.

**Equipment and solutions**

b) **Device:** Prismaflex system (Gambro-Baxter, Deerfield, IL, USA)
c) **Catheter selection:** Venous access was obtained by dual lumen hemodialysis catheters (7 F to 12 F) depending on the age and weight of the child [49].

**Table S2.** Catheter size selection according to body weight in CKRT patients

| Patient weight | Catheter size |
| --- | --- |
| 3-6 kg | 7 French |
| 6-15 kg | 8-9 French |
| 15-30 kg | 9-10 French |
| >30 kg | 10-12 French |

CKRT: continuous kidney replacement therapy

d) **Circuit selection:** Poly aryl ethylene sulphone (PAES) membranes (Prismaflex HF20; circuit volume 60 mL, surface area 0.2 m^2^) or AN69 membranes (Prismaflex M60; circuit volume 93 mL, surface area 0.6 m^2^ and Prismaflex M100; circuit volume 152 mL, surface area 0.9 m^2^) were used. Children with a body weight of <10 kg underwent CKRT using a Prismaflex HF20 set, those with a body weight between 10 kg and 25 kg received the Prismaflex M60 set, while those exceeding 25 kg received a Prismaflex M100 set.
e) **Circuit priming:** Heparinized saline (5 U/mL) was the primary method used for hemofilter priming. In patients with high risk for hemorrhage however, the circuit was primed via the blood-priming method, particularly among children with a bodyweight of less than 10 kg and those with hemoglobin values below 10 g/dL. For this purpose, erythrocyte suspension was diluted 1-to-1 with saline.
f) **Citrate solution:** Prismocitrate 18/0 (Baxter, USA) (contains 140 mmol/L sodium, 18 mmol/L citrate)
g) **Dialysate solution:** Prism0cal B22 (Baxter, USA) (contains 140 mmol/L sodium, 22 mmol/L HCO_3_^-^)
h) **Replacement solution:** Dialisan (Baxter, USA) (contains 140 mmol/L sodium, 2 mmol/L potassium, 32 mmol/L HCO_3_^-^)
i) **Calcium solution:** Citrate effect was neutralized using a continuous calcium infusion, calcium gluconate 10% 50/50 with dextrose 5% (116 mmol/L). Calcium infusion was administered through a different central venous line. In cases where a separate central line was not available, we administered calcium infusion through the return line of the circuit.

**Treatment Monitoring**

a) **Ionized calcium target:** From January 2022 to May 2023, the target pf-iCa^++^ level was maintained between 0.25 and 0.35 mmol/L. After May 2023, the target pf-iCa^++^ level was set between 0.30 and 0.40 mmol/L. The patient’s (systemic) iCa^++^ target was between 1.0 and 1.2 mmol/L. Calcium infusion rate was initiated according to the following formula: citrate flow rate (mL/h) × 0.03. Dose adjustments based on pf-iCa^++^ and patient iCa^++^ levels were made according to Table S3 and Table S4. pf-iCa^++^ and patient iCa^++^ levels were evaluated simultaneously. The monitoring of iCa^++^ concentrations was performed at several time points to ensure maintenance of target concentrations: at the 30th minute, the first, second and fourth hour of therapy, and then routinely every 4 h. Pf-iCa^++^ and patient iCa^++^ levels were also checked one hour after any change in blood flow, citrate concentration, or dialysis prescription.

**Table S3.** Dose adjustment table in patients weighing ≤ 20 kg

| ≤ 20 kg | | Post-filter ionized calcium level (mmol/L) | | | |
| --- | --- | --- | --- | --- | --- |
|  |  | <0.25^*^  <0.30^**^ | 0.25-0.35^*^  0.30-0.40^**^ | 0.35-0.60^*^  0.40-0.60^**^ | >0.6 |
| Patient ionized calcium level (mmol/L) | >1.2 | Flow rate **reduce**  2 mmol/L | **Maintain** citrate flow rate | Citrate flow rate **increase** 0.2 mmol/L | Citrate flow rate **increase** 0.4 mmol/L |
|  |  | Calcium flow rate **reduce** 2.4 mL/h | Calcium flow rate **reduce**  2.4 mL/h | Calcium flow rate  **reduce** 2.4 mL/hour | Calcium flow rate  **reduce** 2.4 mL/hour |
|  | 1-1.2 | Citrate flow rate **reduce** 0.2 mmol/L | **Maintain** citrate flow rate | Citrate flow rate **increase** 0.2 mmol/L | Citrate flow rate **increase** 0.4 mmol/L |
|  |  | **Maintain** calcium flow rate | **Maintain** calcium flow rate | **Maintain** calcium flow rate | **Maintain** calcium flow rate |
|  | 0.9-1 | Citrate flow rate  **reduce** 0.2 mmol/L | **Maintain** citrate flow rate | Citrate flow rate **increase** 0.2 mmol/L | Citrate flow rate **increase** 0.4 mmol/L |
|  |  | Calcium flow rate **increase** 2.4 mL/h | Calcium flow rate **increase** 2.4 mL/hour | Calcium flow rate **increase** 2.4 mL/h | Calcium flow rate **increase** 2.4 mL/h |
|  | <0.9 | Citrate flow rate  **reduce** 0.2 mmol/L | **Maintain** citrate flow rate | Citrate flow rate **increase** 0.2 mmol/L | Citrate flow rate **increase** 0.4 mmol/L |
|  |  | Calcium flow rate **increase** 4.8 mL/h | Calcium flow rate **increase** 4.8 mL/h | Calcium flow rate **increase** 4.8 mL/h | Calcium flow rate **increase** 4.8 mL/h |

*: In the period from January 2022 to May 2023, the target iCa^++^ level was 0.25-0.35 mmol/L, and citrate and calcium infusion dose adjustments were made according to the specified references.

**: In the period after May 2023, the post-filter target iCa^++^ level was 0.30-0.40 mmol/L, and citrate and calcium infusion dose adjustments were made according to the specified references.

**Table S4.** Dose adjustment table in patients weighing >20 kg

| >20 kg | | Post-filter ionized calcium level (mmol/L) | | | |
| --- | --- | --- | --- | --- | --- |
|  |  | <0.25^*^  <0.30^**^ | 0.25-0.35^*^  0.30-0.40^**^ | 0.35-0.60^*^  0.40-0.60^**^ | >0.6 |
| Patient ionized calcium level (mmol/L) | >1.2 | Flow rate **reduce**  2 mmol/L | **Maintain** citrate flow rate | Citrat flow rate **increase** 0.2 mmol/L | Citrat flow rate **increase** 0.4 mmol/L |
|  |  | Calcium flow rate **reduce** 4.8 mL/h | Calcium flow rate **reduce** 4.8 mL/h | Calcium flow rate  **reduce** 4.8mL/h | Calcium flow rate  **reduce 4**.8 mL/h |
|  | 1-1.2 | Citrate flow rate **reduce** 0.2 mmol/L | **Maintain** citrate flow rate | Citrat flow rate **increase** 0.2 mmol/L | Citrat flow rate **increase** 0.4 mmol/L |
|  |  | **Maintain** calcium flow rate | **Maintain** calcium flow rate | **Maintain** calcium flow rate | **Maintain** calcium flow rate |
|  | 0.9-1 | Citrate flow rate  **reduce** 0.2 mmol/L | **Maintain** citrate flow rate | Citrate flow rate **increase** 0.2 mmol/L | Citrate flow rate **increase** 0.4 mmol/L |
|  |  | Calcium flow rate **increase** 4.8 mL/h | Calcium flow rate **increase** 4.8 mL/h | Calcium flow rate **increase** 4.8 mL/h | Calcium flow rate **increase** 4.8 mL/h |
|  | <0.9 | Citrate flow rate  **reduce** 0.2 mmol/L | **Maintain** citrate flow rate | Citrate flow rate **increase** 0.2 mmol/L | Citrate flow rate **increase** 0.4 mmol/L |
|  |  | Calcium flow rate **increase** 9.6 mL/h | Calcium flow rate **increase 9**.6 mL/h | Calcium flow rate **increase** 9.6 mL/h | Calcium flow rate **increase** 9.6 mL/h |

*: In the period from January 2022 to May 2023, the target iCa^++^ level was 0.25-0.35 mmol/L, and citrate and calcium infusion dose adjustments were made according to the specified references.

**: In the period after May 2023, the post-filter target iCa^++^ level was 0.30-0.40 mmol/L, and citrate and calcium infusion dose adjustments were made according to the specified references.

b) **Assessment of citrate-related metabolic and electrolyte disturbance**

Electrolyte-related and metabolic disturbances during CRRT were recorded and categorized as follows: hypocalcemia (iCa^++^ < 1.0 mmol/L), hypercalcemia (iCa^++^ > 1.20 mmol/L), hyponatremia (Na < 130 mmol/L), hypernatremia (Na > 145 mmol/L), hypomagnesemia (< 1.5 mg/dL), hypophosphatemia (< 2.5 mg/dL), metabolic acidosis (pH < 7.35 or base excess [BE] < −3), and metabolic alkalosis (pH > 7.45 or BE > +3). Citrate accumulation was defined as a ratio of total serum calcium (T) to ionized serum calcium (iCa^++^) of > 2.5 (T/iCa^++^ > 2.5) without metabolic acidosis and hypocalcemia [11, 12, 49]. Citrate lock syndrome was defined as ratio of T/iCa^++^ > 2.5 with high anion gap metabolic acidosis [22].
